# Towards Patient-Centered Conflicts of Interest Policy

**DOI:** 10.15171/ijhpm.2017.128

**Published:** 2017-10-29

**Authors:** Peter D. Young, Dawei Xie, Harald Schmidt

**Affiliations:** ^1^Berman Institute of Bioethics, Johns Hopkins University, Baltimore, MD, USA.; ^2^Biostatistics and Epidemiology, Hospital of the University of Pennsylvania, Philadelphia, PA, USA.; ^3^Center for Health Incentives and Behavioral Economics, University of Pennsylvania, Philadelphia, PA, USA.; ^4^Department of Medical Ethics and Health Policy, Perelman School of Medicine at the University of Pennsylvania, Philadelphia, PA, USA.

**Keywords:** Conflict of Interest, Physician-Industry Relationships, Informed Consent, Physician Payments Sunshine Act, Open Payments Program (OPP)

## Abstract

Financial conflicts of interest exist between industry and physicians, and these relationships have the power to influence physicians’ medical practice. Transparency about conflicts matters for ensuring adequate informed consent, controlling healthcare expenditure, and encouraging physicians’ reflection on professionalism. The US Centers for Medicare & Medicaid Services (CMS) launched the Open Payments Program (OPP) to publicly disclose and bring transparency to the relationships between industry and physicians in the United States. We set out to explore user awareness of the database and the ease of accessibility to disclosed information, however, as we show, both awareness and actual use are very low. Two practical policies can greatly enhance its intended function and help alleviate ethical tension. The first is to provide data for individual physicians not merely in absolute terms, but in meaningful context, that is, in relation to the zip code, city, and state averages. The second increases access to the OPP dataset by adding hyperlinks from physicians’ professional websites directly to their Open Payments disclosure pages. These changes considerably improve transparency and the utility of available data, and can furthermore enhance professionalism and accountability by encouraging physicians to reflect more actively on their own practices.

## Background

The importance of disclosing conflicts of interest has been recognized by key international health organizations including the World Medical Association,^1,2^ the World Health Organization (WHO),^3^ the Council for International Organizations of Medical Sciences,^4^ and the Nuffield Council on Bioethics.^5^ Despite international agreement on the need for transparency from leading policy organizations, conflicts of interests (COIs) are still problematic world-wide.^6-11^

To help document interactions between physicians and industry, the United States,^12^ Japan,^13^ Australia,^14^ France,^15^ and more than 30 European countries represented by the European Federation of Pharmaceutical Industries and Associations (EFPIA)^16^ implemented public databases detailing financial interactions between physicians and industry. While this trend of public disclosure is gaining precedence within wealthier countries, similar public databases could become useful in low- and middle-income countries in an effort to strive toward a higher standard of good governance.

Our investigation here assesses the physician-industry COI disclosure database in the United States, known as the Open Payments Program (OPP), by focusing on user awareness of the database and the ease of accessibility to information that impacts health care.

## US Context

In light of a growing concern for COIs in the United States, and in response to the need for more empirical evidence documenting their existence, the US Institutes of Medicine (IOM) released a comprehensive report in 2009 titled Conflict of Interest in Medical Research, Education, and Practice, which found among other things that a physician who has a financial relationship with a third party company, may be influenced to prescribe that company’s medicine even when other therapies are indicated to be more beneficial.^17^

Following the release of IOM report there was a push to pass the Physician Payment Sunshine Act,^18^ which was eventually included as Section 6002 in the Affordable Care Act (ACA).^19^ The Department of Health and Human Services then tasked the Centers for Medicare & Medicaid Services (CMS) to establish a federal database on industry payments to physicians caring for Medicaid, Medicare, and the Children’s Health Insurance Program (CHIP) patients.^20^ This online database, implemented in 2014, came to be known as the OPP,^12^ and it allows users to look up physicians’ names to see the financial relationships those doctors have with industry.

For 2015, the OPP shows that $1.99 billion in general payments and $85.58 million in research payments were made to as many as 618 000 physicians.^21^ The largest single general payment amounted to $30.0 million, the smallest to $0.01. Patients can search the OPP for individual physicians and view their total annual payments, as well as 16 payment subcategories, including consulting and speaking fees, honoraria, gifts, entertainment, food and beverage, travel and lodging, research payments, and company ownership stakes.^22^

### COI Transparency: The Ethical Imperative

COIs are classically conceptualized as “a set of circumstances or conditions in which professional judgment of a primary interest, such as the integrity and quality of research, tends to be unduly influenced by a secondary interest...”^23^ The primary interest of physicians is to promote the health of their patients. Secondary interests are additional goals that could be related to the profession, but may also be completely distinct. They include increasing one’s income, earning grant funding, or being promoted. A conflict of interest is a tendency for secondary interests to be prioritized over primary interests.

Not all industry payments to physicians lead to COIs that negatively affect patients in prescribing or other practices. In principle, industry payments may also have positive effects. For example, certain types of payment from industry can be a marker of physician skill that may translate into better patient care. However, the academic literature as well as court proceedings, clearly demonstrate an undesirable frequency of cases in which physicians’ personal interests override evidence-based diagnostic or therapeutic choices.^17,24,25^ Social scientists have shown that personal benefits can unduly influence decision-making,^26^ and financial COI have been demonstrated to affect the prescribing patterns of physicians.^27-30^

In the worst case of COIs, patients experience harm from medically unnecessary drugs or interventions,^17^ but COIs can also harm patients when medical need is uncontroversial. For example, physicians may choose among different interventions those that benefit themselves the most, even when other interventions are more effective, or differ in side-effects in ways that matter to patients.^29-31^ Furthermore, patients may be harmed financially. Out-of-pocket cost can vary considerably between interventions, and physicians’ choices may needlessly burden patients with a higher share of the cost, for example, where costly branded drugs that confer a financial benefit to the physician are prescribed instead of generics.^31^

For these reasons, chiefly, patients need to know if physicians might be guided by interests other than providing evidence-based care. Consent in clinical practice—a foundational cornerstone of medical ethics—is meaningless where COIs substantially affect the type and amount of information or interventions that patients receive. If there is a shortcoming in the full disclosure of information, the consent given cannot justifiably be considered informed.^32^ Physicians themselves may not always disclose industry payments to patients in consultations, which underlines the ethical importance of the OPP that expressly aims to “permit patients to make better informed decisions when choosing health care professionals.”^20^ In a secondary, yet also important ethical perspective, the OPP further seeks to mitigate negative budgetary impacts. It is hoped that the published data will “deter inappropriate financial relationships which can sometimes lead to increased healthcare costs.”^20^

### The Ethical Policy Response to COI

Despite the findings of the IOM report and other robust data,^17,24,25,33^ some doubt the seriousness of COI in medicine. A recent controversy revealed profound disagreement among leading commentators about whether COI data should be disclosed.^34-37^ But COI policy must not only satisfy experts. First and foremost, it must work for those at risk of dramatic consequences from COIs—patients.

Patients’ perceptions of COI disclosure impact the physician-patient relationship. In general, patients who believe that their physician is receiving gifts or payments from industry, for example, have lower trust in their physicians.^38,39^ The type of payment, however, also matters to patients. Some payment categories, such as consulting, are seen as more acceptable, while others, such as payment for travel and accommodations at a resort, are met with lower approval.^39^

Even if physicians have financial COI with industry, when they disclosed those relationships, patients either trusted them more,^40^ or the level of trust remained the same.^41^ If disclosure is more accessible, it can prompt open conversations about COI between physicians and patients and potentially lead to more trusting relationships and better health outcomes.

COI disclosure has further significance for patient decision-making. A 2012 study shows that patients are less likely to take a prescribed drug if a physician had recently received a gift from the manufacturer in exchange for listening to a pharmaceutical representative speak about that drug.^42^ The authors also found that when patients mistrust their provider, they are less likely to adhere to a prescribed treatment or other recommendations, which could have serious adverse health effects.^42^ Knowing about their physician’s relationship with industry, therefore, has real implications to patients who may change their treatment decisions or involvement in a clinical trial based on COI information.^43^

In principle, the importance of COI disclosure to patient decision-making and physicians’ reflection on their own professional practice has been recognized with the OPP database, which can provide patients and physicians with important information and support ethically meaningful consent. Yet, it is not clear that the database is realizing its full potential.

To assess the scope of improvement, we administered a survey asking US residents if knowing about COI was important to them and whether they were aware of, and had ever accessed, the OPP database. We also ran searches on the OPP database and the portal of a major health plan as a hypothetical patient interested in ascertaining physicians’ COIs, to determine whether COI data were easy to find and understand.

## Methods

### Survey of US Residents’ Attitudes towards COI

We surveyed US residents about the importance of financial COI between healthcare manufacturing companies and physicians. To gain access to a representative sample of Americans, we used GfK Custom Research North America’s KnowledgePanel™. The panel is a probability-based online non-volunteer access panel that combines address-based and random digit dial sampling. It comprises 55 000 adult members and is estimated to cover approximately 97% of US households. Recruited non-internet households receive a laptop computer and free internet service. For each survey, active panel members are drawn using a probability proportional to size (PPS) weighted sampling approach. The Panel’s methodology is otherwise described in detail on the company’s website.^44,45^ GfK’s policies outlining participant voluntariness and informed consent can also be found online.^46^

Our instrument comprised two brief multiple choice questions that were pilot tested with 10 participants. In fielding the survey, the questions were combined with others about heat waves, genetic testing, and future Olympic Games (there were no other healthcare related questions that could have led to framing effects). To further reduce framing effects, we also alternated the sequence of answers. The findings contained in this report are based on a survey fielded from June 19–21, 2015. As per GFK policy, the outgoing sample was PPS weighted, and the incoming sample had further (proprietary) demographic weights applied to ensure representativeness. The outgoing sample comprised 3125 individuals, and the incoming sample of 1005, was made up of 510 male and 495 female adults, all 18 years of age and over. This gave a completion rate of 34.7%, although the rate cannot be seen as indicating a particular willingness or lack thereof in terms of responding to questions about COIs, as questions were bundled with the above noted, unrelated ones.

### Statistical Analysis

We analysed the distribution for each variable after GfK’s weighting using SAS 9.4 statistical analysis software. In our analysis, we included variables for gender, age, income, region, race, education, marital status, employment status, own or rent home, housing type, and households with internet access. We then dichotomized responses into two categories, unimportant (containing “completely unimportant” and “not very important”) and important (containing “somewhat important” and “very important”). Participants who refused to answer were treated as missing. Since there was no obvious hypothesis that a particular variable would be central, we looked at the association between each of the demographic variables with the dichotomized variable, adding all the variables to a logistic regression model, assuming an alpha level of 5% when determining statistical significance.

### Search Strategy for Health Plan Data

We adopted the perspective of a hypothetical patient looking for a cardiovascular disease specialist in the randomly determined zip code of 44106, with Blue Cross Blue Shield’s Medicare Advantage PPO (Preferred Provider Organization) coverage. This search was performed on October 21, 2015.

We accessed the health plan’s database via the portal found at: http://provider.bcbs.com/. Once there, we selected the ‘Medicare Advantage PPO’ network and the ‘Ohio, Anthem Blue Cross and Blue Shield’ plan. We then specified ‘cardiovascular disease’ for the specialty and ‘44106’ for the zip code. A total of 21 cardiovascular disease specialists were covered by ‘Ohio, Anthem Blue Cross and Blue Shield’ for the 44106 zip code.

### Search Strategy for Open Payments Program Data

We then mirrored the steps that a patient would take to determine whether doctors identified in the Health Plan Search were listed on the OPP database, again looking for cardiovascular disease specialists practicing in the randomly determined zip code of 44106. This search was performed on October 21, 2015.

We accessed the OPP at: https://openpaymentsdata.cms.gov/search, and entered ‘44106’ for the zip code and ‘Allopathic & Osteopathic Physicians/Internal Medicine, Cardiovascular Disease’ for specialty. We searched only for these two parameters. A list of 31 physicians’ names were retrieved for this specialty and zip code.

To find the total payments received in 2014, we went to each physician’s summary page in the OPP. In our study, ‘Total Payment Amount’ for each physician was aggregated from fields “Total General Payments,” “Total Research Payments,” and “Total Associated Research Funding.” We determined the median payment amount for physicians found exclusively in the OPP database. Separately, we determined the median payment amount for physicians found in both the OPP database and Blue Cross Blue Shield insurance portal. Physicians found in the Blue Cross Blue Shield portal but not the OPP database were assumed to have received no money ($0.00) from third party companies in 2014.

## Results

### Survey Results—Open Payments Program’s Potential

Which payments matter to patients? In our nationally representative survey of 1005 US residents age 18 and older, we found that while roughly one-third of respondents appeared to not care about transparency, even when—as described in our prompt—possible bias in physician prescribing can occur, knowing about benefits such as dinners at fine restaurants or paid lectures was important/very important to 63.5% of respondents (Table 1).

**Table 1:** The Importance of Physician-Industry COI Disclosure According to US Residents

	**Total**	**(Percent)**
Total weighted	1000	(100.0)
Completely unimportant/Not very important (Net)	359	(35.9)
Completely unimportant	80	(8.0)
Not very important	279	(27.9)
Somewhat important/Very important (Net)	635	(63.5)
Somewhat important	400	(40.0)
Very important	234	(23.4)
Refused	6	(0.6)

Abbriviation: COI, conflicts of interest. US residents, 18 years and older, were informed that “Pharmaceutical and other health care manufacturing companies often promote their drugs and other products by inviting physicians to free dinners at fine restaurants. Companies also pay physicians for talks or lectures. Studies have shown that as a result, physicians can be more likely to prescribe the companies’ products.” Following this, they were asked, “In choosing a physician: how important is it to you to know whether your physician has received such benefits?” Scale was shown on a rotating alternating basis. The margin of error on weighted data is ±3 percentage points for the full sample.

We looked at the association between each variable with the dichotomized importance variable and found there was no significant association. The association remained insignificant when we added all the variables to a logistic regression model at the same time (see Supplementary file 1).

In principle, the OPP is therefore of considerable use—but we also found that only 7.9% were aware of it, and a mere 1.5% had ever accessed it (Table 2).

**Table 2:** Whether US Residents Have Heard of or Used the CMS Open Payments Database

	**Total**	**(Percent)**
Total weighted	1000	(100.0)
I have used the CMS Open Payment database.	15	(1.5)
I have heard of the CMS Open Payment database but never used it.	79	(7.9)
I have never heard of the CMS Open Payment Database.	896	(89.6)
Refused	9	(0.9)

Abbreviation: CMS, Centers for Medicare & Medicaid Services. US residents, 18 years and older, were asked, “How aware are you of the CMS Open Payments Database that can be searched on the internet by anyone to learn about financial relationships that particular physicians have with health care manufacturing companies?” Scale was shown on a rotating alternating basis. The margin of error on weighted data is ±3 percentage points for the full sample.

### Open Payments Program and Blue Cross Blue Shield Data

Information in the OPP is not provided as helpfully as it could be. In a significant sense, the OPP is abstract and does not dovetail easily with the way patients go about finding a doctor when they need one. These searches are typically constrained by what choices physician networks and health care plans make available to patients; which physicians, among these, see patients in an appropriate time frame; and among these, which are within a reasonable distance. The following example, based on the searches described above, illustrates this.

Suppose you are a Cleveland-based patient requiring a cardiovascular disease specialist. Your Blue Cross Blue Shield insurance portal identifies 21 doctors in your zip code (44106). You review their credentials on their professional websites, and search the same criteria within the OPP. The OPP shows the first physician received a total of $12 in 2014. The second, $110 472. You find another 29 physicians in this specialty and zip code, although eight physicians from your insurance website cannot be found in the OPP database, which you presume means that they did not accept industry payments. You calculate that the median annual payment for the Blue Cross Blue Shield identified physicians in this specialty and zip code is $379, and $9203 for only those listed in the OPP (Table 3). This puts the individual payments somewhat into perspective—even though you do not know how physicians compare to the city or state averages. And comparisons across particular payment subcategories (gifts, dining, speaker’s fees, etc) would require further manual searches.

**Table 3:** The Value of Comparative Data Output: Comparing Total Payment Amounts Received in 2014 by Cardiac Disease Specialists Who Practice Within the 44106 Zip Code

#	**Physician'sInitials**	**Is the Physician Listed on the Blue Cross Blue Shield Website?**	**Is the Physician Listed in the Open Payments Database?**	**Total Payment Amount for Physicians Listed in OPP**	**Total Payment Amount Modified to Include Physicians not Listed in OPP**
1	M.A.*	X			$0.00
2	A.P.*	X			$0.00
3	N.R.*	X			$0.00
4	D.S.*	X			$0.00
5	J.S.*	X			$0.00
6	R.W.	X			$0.00
7	T.W.*	X			$0.00
8	W.W.	X			$0.00
9	S.H.		X	$11.81	$11.81
10	R.N.		X	$14.25	$14.25
11	D.S.		X	$46.03	$46.03
12	R.J.	X	X	$67.31	$67.31
13	L.G.	X	X	$136.61	$136.61
14	C.L.	X	X	$150.91	$150.91
15	T.J.	X	X	$209.15	$209.15
16	J.K.		X	$231.87	$231.87
17	A.H.		X	$271.59	$271.59
18	J.S.	X	X	$344.99	$344.99
19	C.A.	X	X	$358.24	$358.24
20	C.B.		X	$379.36	$379.36
21	M.G.	X	X	$427.68	$427.68
22	M.F.		X	$585.39	$585.39
23	V.N.		X	$1205.88	$1205.88
24	M.C.	X	X	$9203.45	$9203.45
25	G.A.	X	X	$11 460.03	$11 460.03
26	A.I.		X	$11 852.27	$11 852.27
27	J.G.		X	$14 225.07	$14 225.07
28	G.O.	X	X	$18 053.60	$18 053.60
29	T.L.		X	$18 106.21	$18 106.21
30	F.S.		X	$20 762.44	$20 762.44
31	D.S.	X	X	$25 904.02	$25 904.02
32	B.E.		X	$37 359.23	$37 359.23
33	H.M.	X	X	$37 905.03	$37 905.03
34	B.H.	X	X	$62 987.91	$62 987.91
35	S.P.		X	$75 623.91	$75 623.91
36	J.O.		X	$83 804.46	$83 804.46
37	D.Z.		X	$88 827.86	$88 827.86
38	H.B.		X	$110 472.28	$110 472.28
39	M.C.		X	$447 507.92	$447 507.92
			Median	$9203.45	$379.36

Abbreviation: OPP, Open Payments Program. We searched for ‘cardiovascular disease’ specialists covered under the Medicare Advantage PPO’ insurance plan in the Blue Cross Blue Shield web portal who also practice within the 44106 zip code. This search yielded 21 physicians. We also searched for cardiovascular disease specialists in the OPP database who practice within the 44106 zip code. By the nature of this search, the 29 OPP-listed physicians received benefits from pharmaceutical or device companies in 2014. Both searches were completed on October 21, 2015. Physicians with an asterisk (*) were listed in the OPP database under a different specialty.

## Discussion

Data listed in the OPP are organized suboptimally, and there is very low public awareness about the program. The result is transparency in obscurity, at best. A survey conducted in 2014 independently from ours paints a similar picture, finding that 12% of US citizens knew payment information was publically available, with 5% knowing whether their physicians received payments.^47^

Two simple policy improvements can substantially improve transparency, the validity of patients’ consent, and physicians’ reflection on COIs: meaningful contextualization of payment data and linking to physicians’ database entries directly from professional portals.

### Improvements to Open Payments Program

Big data do not automatically translate into big benefits. But small tweaks can make big differences. For example, ProPublica’s Dollars for Docs database renders the OPP dataset to rank each physician by annual total payments within specialty in relation to state and national averages.^48^ The denominator here ignores physicians without records and comparisons hence understate the significance of payments. But the approach nonetheless provides some context, and illustrates the technical feasibility of implementing comparative data output. In a similar vein, recently, the OPP was redesigned to provide national and specialty means.

While these most recent revisions should be welcomed, they also need to go further and enable comparisons not just be provided nationally, but within states, and by city and zip code limits—the more typical boundaries for patients’ physician searches.

Dollar amounts alone could be meaningless to patients. But comparisons between all physicians a patient is considering, and with similar specialists in other locations can better orientate patients in selecting physicians. To be clear, high payments need not rule out physicians, but awareness can enable patients to raise the role of payments in the initial consultation to ensure care is not compromised.

### Open Payments Program Access Improvements

Better organization of data are necessary, but not sufficient for meaningful OPP use. It is imperative to improve OPP access. Organizations such as the Patient-Centered Outcomes Research Institute (PCORI), or the US Preventive Services Task Force list conflict of interest information along with other biographical data.^49,50^ For context considered here, the most appropriate strategy would embed OPP data in patients’ typical searches by simply adding hyperlinks on physicians’ professional websites to their OPP entries. Physicians’ websites typically list qualifications, certifications, publications, and sometimes grants. COI data may be just as—if not more—relevant to patients.^42^ Hospitals, health systems, and professional organizations usually specify website content categories. There is therefore considerable potential to demonstrate leadership in furthering patient-centered COI policy by providing patients with ready access to COI data by linking directly to the OPP.

### Improving Physicians’ Reflections on COI

Enhanced COI transparency may also change physicians’ behavior, as two analogies illustrate. Regarding electricity usage, high energy users who were made aware of their neighbors’ lower usage eventually decreased their own.^51^ COI peer-comparisons could plausibly have similar effects. Physicians may not view their behavior as exceptional if immediate colleagues receive similar benefits. But if data clearly show their behavior as significant outliers at the zip code, city, or state level, the appropriateness of accepting benefits may be reconsidered.

The fact that comparisons are public can provide a further and independent incentive. For example, in advance of the Affordable Care Act’s requirement for large US chain restaurants to post calories, major companies reduced calories by around 12% for newly introduced items.^52^ The effect of what might be called the “benevolent pillory” recognizes that perceptions of appropriate behavior can be as powerful as substantive agreement, and this can provide additional leverage in protecting patients from COIs.

Recent empirical work supports the plausibility of these arguments. In a series of six focus groups with 42 physicians, Chimonas and colleagues found that physicians have used the OPP database to reflect on their own payments and to check payments received by colleagues. Physicians welcomed the idea of transparency, but they had a broad unawareness of and lack of understanding about COIs.^53^

## Limitations

For the example of a Cleveland-based patient requiring a cardiovascular disease specialist, we used the randomly selected zip code of 44106, a suburb of Cleveland, Ohio, which is also in close proximity to two large academic medical centers, University hospitals and the Cleveland Clinic. We also only looked at a single zip code, which is not likely representative of much of the United States. We took this approach to illustrate the basic problem, not to give a sense of how typical this spread is. We also did not collect qualitative data from actual patients on how easy or difficult it is to navigate the OPP online portal. Our aim was to show the type quantitative data that can be retrieved by patient searching for a particular physician and zip code.

We proposed that comparing providers within a geographic region is a worthwhile improvement to be made in presenting OPP data. It is important to appreciate that even if this is implemented, interpretation of these data can be confounded by other factors, for example, whether the providers are in academic versus private practice; the fact that certain types of payments, such as consulting,^39^ can be viewed as favorable; or that medians and means side-by-side can convey different messages. These and other related issues could be addressed either through more sophisticated data presentations or brief explanations—even if both impact the ease of use and intelligibility to lay users. Empirical testing can reveal which further tweaks are desirable, but overall, the perfect should not be the enemy of the good, and we maintain that significant progress can be made by implementing the proposals we suggest here.

## Conclusion

COIs risk undercutting the principle of informed consent with the potential to harm patients physically, psychologically, and financially. COIs can also needlessly increase healthcare cost. Truly patient-centerd COI policy requires contextualized data visualizations and modes of access that align naturally with the way patients search for physicians. While there is still much work to be done with collecting payment data and deciding as a society how to react to them, our two suggestions—providing data for individual physicians in relation to zip code, city, and state averages, and creating hyperlinks from physicians’ professional websites directly to their Open Payments disclosure pages—are simple in their implementation, and could go a long way in helping patients and the medical community as a whole. While knowing if a COI exists by itself alone will not make the difference between informed and uninformed consent, it seems hard to see on what grounds it should be acceptable to deny patients the opportunity to integrate data on possible conflicts in their decision-making—especially since these have been made available in principle.

## Ethical issues

Since participation did not expose respondents to risks, and since data were gathered anonymously and reported in the aggregate only, IRB review was not required.

## Competing interests

This work was not sponsored by a funder with proprietary or financial interests in the outcome.

## Authors’ contributions

PDY and HS contributed to the conception and design of the study, and both wrote the discussion. PDY, HS, and DX analysed and interpreted the data. DX performed the statistical analysis.

## Authors’ affiliations

^1^Berman Institute of Bioethics, Johns Hopkins University, Baltimore, MD, USA. ^2^Biostatistics and Epidemiology, Hospital of the University of Pennsylvania, Philadelphia, PA, USA. ^3^Center for Health Incentives and Behavioral Economics, University of Pennsylvania, Philadelphia, PA, USA. ^4^Department of Medical Ethics and Health Policy, Perelman School of Medicine at the University of Pennsylvania, Philadelphia, PA, USA.

## Supplementary files

Supplementary files Supplementary file 1 contains statistical analysis.
