# Supplementary material for: Towards Patient-Centered Conflicts of Interest Policy
Source: Int J Health Policy Manag. 2017 Oct 29;7(2):112–9. doi: 10.15171/ijhpm.2017.128 (PMC5819371; doi:10.15171/ijhpm.2017.128)
Supplement: Supplementary files — Supplementary file 1 contains statistical analysis. [file ijhpm-7-112-s001.pdf]

Statistical Analysis

Methods:

Q1 was recorded assuming it is the question asking about the importance of physician-industry disclosure to unimportant (1 and 2) and important (3 and 4). The refused are treated as missing.

A 4-category variable was created for age, and a 4-category variable was created for income. The categories are shown in the attached results. The results contain the following output:

1. Pages 1-3 provide the unweighted distribution of each variable. Also included are other variables such as race, education, marital status, employment status, own or rent home, housing type, and households with internet access.
2. Pages 4-6 show the weighted distribution of these variables.
3. Pages 7-17 shows the weighted two-way crosstab tables for each variable and the dichotomous Q1 variable. None of the variables are significantly associated with Q1.
4. Pages 18-21 show the logistic regression (weighted) with the dichotomous Q1 variable as the outcome and gender, age, income and region in the model. None is significant and other variables were not added because none of them are significant in step 3.

q1_dummy	Frequency	Percent	Cumulative Frequency	Cumulative Percent
important	633	63.30	633	63.30
unimportant	367	36.70	1000	100.00
Frequency Missing = 5				

PPGENDER Sex of respondent				
ppgender	Frequency	Percent	Cumulative Frequency	Cumulative Percent
male	510	50.75	510	50.75
female	495	49.25	1005	100.00

age4	Frequency	Percent	Cumulative Frequency	Cumulative Percent
18-34	322	32.04	322	32.04
35-49	234	23.28	556	55.32
50-64	295	29.35	851	84.68
65+	154	15.32	1005	100.00

income4	Frequency	Percent	Cumulative Frequency	Cumulative Percent
<$25K	145	14.43	145	14.43
$25-49.9K	209	20.80	354	35.22
$50-74.9K	207	20.60	561	55.82
$75K+	444	44.18	1005	100.00

region	Frequency	Percent	Cumulative Frequency	Cumulative Percent
Northeast	172	17.11	172	17.11
Midwest	243	24.18	415	41.29
South	354	35.22	769	76.52
West	236	23.48	1005	100.00

PPETHM Race				
ppethm	Frequency	Percent	Cumulative Frequency	Cumulative Percent
White, Non-Hispanic	735	73.13	735	73.13
Black, Non-Hispanic	76	7.56	811	80.70
Other, Non-Hispanic	42	4.18	853	84.88
Hispanic	110	10.95	963	95.82
2+ Races, Non-Hispanic	42	4.18	1005	100.00

PPEDUC Education				
ppeduc	Frequency	Percent	Cumulative Frequency	Cumulative Percent
Less than HS graduate	97	9.65	97	9.65
HS grad	284	28.26	381	37.91
Some college, no degree	195	19.40	576	57.31
Associate degree	85	8.46	661	65.77
Bachelor degree	197	19.60	858	85.37
Master degree	113	11.24	971	96.62
Professional or Doctorate degree	34	3.38	1005	100.00

PPMARIT Marital status				
ppmarit	Frequency	Percent	Cumulative Frequency	Cumulative Percent
Married	541	53.83	541	53.83
Widowed	33	3.28	574	57.11
Divorced	86	8.56	660	65.67
Separated	13	1.29	673	66.97
Never married	266	26.47	939	93.43
Living with partner	66	6.57	1005	100.00

PPWORK Employment Status				
ppwork	Frequency	Percent	Cumulative Frequency	Cumulative Percent
Working-as a paid employee	535	53.23	535	53.23
Working-self-employed	69	6.87	604	60.10
Not working - on temporary layoff from a job	7	0.70	611	60.80
Not working - looking for work	82	8.16	693	68.96
Not working - retired	145	14.43	838	83.38
Not working - disabled	59	5.87	897	89.25
Not working - other	108	10.75	1005	100.00

PPRENT Own or rent home				
pprent	Frequency	Percent	Cumulative Frequency	Cumulative Percent
Owned or being bought by you or someone in your household	714	71.04	714	71.04
Rented for cash	258	25.67	972	96.72
Occupied without payment of cash rent	33	3.28	1005	100.00

PPHOUSE Housing type				
pphouse	Frequency	Percent	Cumulative Frequency	Cumulative Percent
A one-family house detached from any other house	732	72.84	732	72.84
A one-family house attached to one or more houses	64	6.37	796	79.20
A building with 2 or more apartments	164	16.32	960	95.52
A mobile home	41	4.08	1001	99.60
Boat/RV/van etc.	4	0.40	1005	100.00

PPNET HHs with Internet Access				
ppnet	Frequency	Percent	Cumulative Frequency	Cumulative Percent
Yes	829	82.49	829	82.49
No	176	17.51	1005	100.00

q1_dummy	Frequency	Percent	Cumulative Frequency	Cumulative Percent
**important**	634.7643	63.85	634.7643	63.85
**unimportant**	359.3343	36.15	994.0987	100.00
**Frequency Missing = 5.9011999965**				

PPGENDER Sex of respondent				
ppgender	Frequency	Percent	Cumulative Frequency	Cumulative Percent
**male**	484.061	48.41	484.061	48.41
**female**	515.9389	51.59	999.9999	100.00

age4	Frequency	Percent	Cumulative Frequency	Cumulative Percent
**18-34**	296.7177	29.67	296.7177	29.67
**35-49**	226.0318	22.60	522.7495	52.27
**50-64**	290.1755	29.02	812.925	81.29
**65+**	187.0749	18.71	999.9999	100.00

income4	Frequency	Percent	Cumulative Frequency	Cumulative Percent
**<$25K**	179	17.90	179	17.90
**$25-49.9K**	225	22.50	403.9999	40.40
**$50-74.9K**	184	18.40	587.9999	58.80
**$75K+**	411.9999	41.20	999.9999	100.00

region	Frequency	Percent	Cumulative Frequency	Cumulative Percent
**Northeast**	182.9905	18.30	182.9905	18.30
**Midwest**	213.9907	21.40	396.9812	39.70
**South**	373.9388	37.39	770.92	77.09
**West**	229.0799	22.91	999.9999	100.00

PPETHM Race				
**ppethm**	**Frequency**	**Percent**	**Cumulative Frequency**	**Cumulative Percent**
**White, Non-Hispanic**	657.0694	65.71	657.0694	65.71
**Black, Non-Hispanic**	115.9814	11.60	773.0508	77.31
**Other, Non-Hispanic**	62.99878	6.30	836.0496	83.60
**Hispanic**	151.9437	15.19	987.9933	98.80
**2+ Races, Non-Hispanic**	12.00658	1.20	999.9999	100.00

PPEDUC Education				
**ppeduc**	**Frequency**	**Percent**	**Cumulative Frequency**	**Cumulative Percent**
**Less than HS graduate**	124.0375	12.40	124.0375	12.40
**HS grad**	296.0044	29.60	420.0419	42.00
**Some college, no degree**	199.5967	19.96	619.6386	61.96
**Associate degree**	88.43436	8.84	708.0729	70.81
**Bachelor degree**	163.7655	16.38	871.8384	87.18
**Master degree**	100.7999	10.08	972.6384	97.26
**Professional or Doctorate degree**	27.36151	2.74	999.9999	100.00

PPMARIT Marital status				
**ppmarit**	**Frequency**	**Percent**	**Cumulative Frequency**	**Cumulative Percent**
**Married**	524.9136	52.49	524.9136	52.49
**Widowed**	45.36483	4.54	570.2784	57.03
**Divorced**	94.59633	9.46	664.8748	66.49
**Separated**	16.46967	1.65	681.3444	68.13
**Never married**	256.1972	25.62	937.5416	93.75
**Living with partner**	62.45825	6.25	999.9999	100.00

PPWORK Employment Status				
**ppwork**	**Frequency**	**Percent**	**Cumulative Frequency**	**Cumulative Percent**
**Working-as a paid employee**	495.3614	49.54	495.3614	49.54
**Working-self-employed**	62.70955	6.27	558.071	55.81
**Not working - on temporary layoff from a job**	6.58408	0.66	564.6551	56.47
**Not working - looking for work**	87.96082	8.80	652.6159	65.26
**Not working - retired**	170.8433	17.08	823.4592	82.35
**Not working - disabled**	68.96151	6.90	892.4207	89.24
**Not working - other**	107.5792	10.76	999.9999	100.00

PPRENT Own or rent home				
**pprent**	**Frequency**	**Percent**	**Cumulative Frequency**	**Cumulative Percent**
**Owned or being bought by you or someone in your household**	694.7943	69.48	694.7943	69.48
**Rented for cash**	269.7932	26.98	964.5875	96.46
**Occupied without payment of cash rent**	35.41234	3.54	999.9999	100.00

PPHOUSE Housing type				
**pphouse**	**Frequency**	**Percent**	**Cumulative Frequency**	**Cumulative Percent**
**A one-family house detached from any other house**	708.1382	70.81	708.1382	70.81
**A one-family house attached to one or more houses**	67.5056	6.75	775.6438	77.56
**A building with 2 or more apartments**	174.3381	17.43	949.9819	95.00
**A mobile home**	45.21339	4.52	995.1953	99.52
**Boat/RV/van etc.**	4.80462	0.48	999.9999	100.00

PPNET HHs with Internet Access				
**ppnet**	**Frequency**	**Percent**	**Cumulative Frequency**	**Cumulative Percent**
**Yes**	785.7531	78.58	785.7531	78.58
**No**	214.2467	21.42	999.9999	100.00

Table of ppgender by q1_dummy			
ppgender(PPGENDER Sex of respondent)	q1_dummy		
Frequency<br>Row Pct	important	unimportant	Total
male	304.346<br>63.29	176.565<br>36.71	480.911
female	330.418<br>64.39	182.77<br>35.61	513.188
Total	634.764	359.334	994.099
Frequency Missing = 5.9011999965			

Statistics for Table of ppgender by q1_dummy

Statistic	DF	Value	Prob
Chi-Square	1	0.1302	0.7183
Likelihood Ratio Chi-Square	1	0.1301	0.7183
Continuity Adj. Chi-Square	1	0.0869	0.7682
Mantel-Haenszel Chi-Square	1	0.1300	0.7184
Phi Coefficient		-0.0114	
Contingency Coefficient		0.0114	
Cramer's V		-0.0114	

Effective Sample Size = 994.09868015
Frequency Missing = 5.9011999965

Table of age4 by q1_dummy			
age4	q1_dummy		
Frequency<br>Row Pct	important	unimportant	Total
18-34	176.727<br>60.01	117.76<br>39.99	294.488
35-49	146.211<br>65.44	77.233<br>34.56	223.444
50-64	188.171<br>65.09	100.921<br>34.91	289.093
65+	123.655<br>66.10	63.4199<br>33.90	187.075
Total	634.764	359.334	994.099
Frequency Missing = 5.9011999965			

Statistics for Table of age4 by q1_dummy

Statistic	DF	Value	Prob
Chi-Square	3	2.7256	0.4359
Likelihood Ratio Chi-Square	3	2.7056	0.4393
Mantel-Haenszel Chi-Square	1	1.9648	0.1610
Phi Coefficient		0.0524	
Contingency Coefficient		0.0523	
Cramer's V		0.0524	

Effective Sample Size = 994.09868015
Frequency Missing = 5.9011999965

Table of income4 by q1_dummy			
income4	q1_dummy		
Frequency<br>Row Pct	important	unimportant	Total
<$25K	114.367<br>65.29	60.7993<br>34.71	175.166
$25-49.9K	144.591<br>64.65	79.0525<br>35.35	223.643
$50-74.9K	113.761<br>62.07	69.5282<br>37.93	183.289
$75K+	262.046<br>63.60	149.954<br>36.40	412
Total	634.764	359.334	994.099
Frequency Missing = 5.9011999965			

Statistics for Table of income4 by q1_dummy

Statistic	DF	Value	Prob
Chi-Square	3	0.4833	0.9225
Likelihood Ratio Chi-Square	3	0.4827	0.9227
Mantel-Haenszel Chi-Square	1	0.2037	0.6517
Phi Coefficient		0.0221	
Contingency Coefficient		0.0220	
Cramer's V		0.0221	

Effective Sample Size = 994.09868015
Frequency Missing = 5.9011999965

Table of region by q1_dummy			
**region**	**q1_dummy**		
**Frequency<br>Row Pct**	**important**	**unimportant**	**Total**
**Northeast**	112.732<br>61.61	70.2582<br>38.39	182.99
**Midwest**	138.596<br>65.73	72.2446<br>34.27	210.841
**South**	235.16<br>63.35	136.027<br>36.65	371.188
**West**	148.276<br>64.73	80.8042<br>35.27	229.08
**Total**	634.764	359.334	994.099
**Frequency Missing = 5.9011999965**			

Statistics for Table of region by q1_dummy

Statistic	DF	Value	Prob
**Chi-Square**	3	0.8399	0.8399
**Likelihood Ratio Chi-Square**	3	0.8392	0.8401
**Mantel-Haenszel Chi-Square**	1	0.1615	0.6878
**Phi Coefficient**		0.0291	
**Contingency Coefficient**		0.0291	
**Cramer's V**		0.0291	

Effective Sample Size = 994.09868015
Frequency Missing = 5.9011999965

Table of ppethm by q1_dummy			
ppethm(PPETHM Race)	q1_dummy		
Frequency<br>Row Pct	important	unimportant	Total
White, Non-Hispanic	403.346<br>61.64	251.057<br>38.36	654.402
Black, Non-Hispanic	78.7736<br>67.92	37.2077<br>32.08	115.981
Other, Non-Hispanic	39.4285<br>62.59	23.5702<br>37.41	62.9988
Hispanic	103.402<br>69.53	45.3079<br>30.47	148.71
2+ Races, Non-Hispanic	9.81472<br>81.74	2.19186<br>18.26	12.0066
Total	634.764	359.334	994.099
Frequency Missing = 5.9011999965			

Statistics for Table of ppethm by q1_dummy

Statistic	DF	Value	Prob
Chi-Square	4	6.0121	0.1982
Likelihood Ratio Chi-Square	4	6.2582	0.1807
Mantel-Haenszel Chi-Square	1	4.3899	0.0362
Phi Coefficient		0.0778	
Contingency Coefficient		0.0775	
Cramer's V		0.0778	

Effective Sample Size = 994.09868015
Frequency Missing = 5.9011999965

Table of ppeduc by q1_dummy			
ppeduc(PPEDUC Education)	q1_dummy		
Frequency<br>Row Pct	important	unimportant	Total
Less than HS graduate	74.0026<br>60.58	48.1575<br>39.42	122.16
HS grad	193.128<br>65.90	99.9359<br>34.10	293.063
Some college, no degree	131.569<br>66.28	66.945<br>33.72	198.514
Associate degree	53.9944<br>61.06	34.4399<br>38.94	88.4344
Bachelor degree	106.335<br>64.93	57.4303<br>35.07	163.766
Master degree	59.6623<br>59.19	41.1376<br>40.81	100.8
Professional or Doctorate degree	16.0734<br>58.74	11.2881<br>41.26	27.3615
Total	634.764	359.334	994.099
Frequency Missing = 5.9011999965			

Statistics for Table of ppeduc by q1_dummy

Statistic	DF	Value	Prob
Chi-Square	6	3.2464	0.7773
Likelihood Ratio Chi-Square	6	3.2251	0.7801
Mantel-Haenszel Chi-Square	1	0.4300	0.5120
Phi Coefficient		0.0571	
Contingency Coefficient		0.0571	
Cramer's V		0.0571	

Effective Sample Size = 994.09868015
Frequency Missing = 5.9011999965

Table of ppmarit by q1_dummy			
ppmarit(PPMARIT Marital status)	q1_dummy		
Frequency<br>Row Pct	important	unimportant	Total
**Married**	322.385<br>61.72	199.94<br>38.28	522.326
**Widowed**	32.065<br>70.68	13.2999<br>29.32	45.3648
**Divorced**	68.1236<br>72.02	26.4728<br>27.98	94.5963
**Separated**	12.3055<br>74.72	4.16419<br>25.28	16.4697
**Never married**	159.07<br>62.63	94.8973<br>37.37	253.967
**Living with partner**	40.8154<br>66.50	20.56<br>33.50	61.3754
**Total**	634.764	359.334	994.099
Frequency Missing = 5.9011999965			

Statistics for Table of ppmarit by q1_dummy

Statistic	DF	Value	Prob
**Chi-Square**	5	5.8676	0.3193
**Likelihood Ratio Chi-Square**	5	6.0469	0.3017
**Mantel-Haenszel Chi-Square**	1	0.4676	0.4941
**Phi Coefficient**		0.0768	
**Contingency Coefficient**		0.0766	
**Cramer's V**		0.0768	

Effective Sample Size = 994.09868015
Frequency Missing = 5.9011999965

Table of ppwork by q1_dummy			
ppwork(PPWORK Employment Status)	q1_dummy		
Frequency<br>Row Pct	important	unimportant	Total
**Working-as a paid employee**	308.346<br>62.58	184.348<br>37.42	492.694
**Working-self-employed**	44.3756<br>70.76	18.334<br>29.24	62.7096
**Not working - on temporary layoff from a job**	5.38224<br>81.75	1.20184<br>18.25	6.58408
**Not working - looking for work**	59.6033<br>68.82	27.0009<br>31.18	86.6043
**Not working - retired**	107.783<br>63.09	63.0606<br>36.91	170.843
**Not working - disabled**	42.7957<br>62.06	26.1658<br>37.94	68.9615
**Not working - other**	66.479<br>62.89	39.2228<br>37.11	105.702
**Total**	634.764	359.334	994.099
**Frequency Missing = 5.9011999965**			

Statistics for Table of ppwork by q1_dummy

Statistic	DF	Value	Prob
**Chi-Square**	6	3.6633	0.7221
**Likelihood Ratio Chi-Square**	6	3.8275	0.7000
**Mantel-Haenszel Chi-Square**	1	0.0037	0.9515
**Phi Coefficient**		0.0607	
**Contingency Coefficient**		0.0606	
**Cramer's V**		0.0607	

Effective Sample Size = 994.09868015
Frequency Missing = 5.9011999965

Table of pprent by q1_dummy			
pprent(PPRENT Own or rent home)	q1_dummy		
Frequency<br>Row Pct	important	unimportant	Total
**Owned or being bought by you or someone in your household**	444.487<br>64.35	246.283<br>35.65	690.77

**Rented for cash**	168.416<br>62.86	99.4996<br>37.14	267.916
**Occupied without payment of cash rent**	21.8607<br>61.73	13.5517<br>38.27	35.4123
**Total**	634.764	359.334	994.099
**Frequency Missing = 5.9011999965**			

Statistics for Table of pprent by q1_dummy

Statistic	DF	Value	Prob
**Chi-Square**	2	0.2560	0.8798
**Likelihood Ratio Chi-Square**	2	0.2552	0.8802
**Mantel-Haenszel Chi-Square**	1	0.2546	0.6139
**Phi Coefficient**		0.0160	
**Contingency Coefficient**		0.0160	
**Cramer's V**		0.0160	

Effective Sample Size = 994.09868015
Frequency Missing = 5.9011999965

Table of pphouse by q1_dummy			
**pphouse(PPHOUSE Housing type)**	**q1_dummy**		
**Frequency<br>Row Pct**	**important**	**unimportant**	**Total**
**A one-family house detached from any other house**	452.114<br>64.38	250.123<br>35.62	702.237
**A one-family house attached to one or more houses**	41.2287<br>61.07	26.2769<br>38.93	67.5056
**A building with 2 or more apartments**	115.049<br>65.99	59.2887<br>34.01	174.338
**A mobile home**	25.2777<br>55.91	19.9357<br>44.09	45.2134
**Boat/RV/van etc.**	1.09507<br>22.79	3.70955<br>77.21	4.80462
**Total**	634.764	359.334	994.099
**Frequency Missing = 5.9011999965**			

Statistics for Table of pphouse by q1_dummy

Statistic	DF	Value	Prob
Chi-Square	4	5.4028	0.2484
Likelihood Ratio Chi-Square	4	5.2359	0.2639
Mantel-Haenszel Chi-Square	1	0.8052	0.3695
Phi Coefficient		0.0737	
Contingency Coefficient		0.0735	
Cramer's V		0.0737	

Effective Sample Size = 994.09868015
Frequency Missing = 5.9011999965

Table of ppnet by q1_dummy			
ppnet(PPNET HHs with Internet Access)	q1_dummy		
Frequency<br>Row Pct	important	unimportant	Total
Yes	507.966<br>64.71	277.077<br>35.29	785.042
No	126.799<br>60.65	82.2574<br>39.35	209.056
Total	634.764	359.334	994.099
Frequency Missing = 5.9011999965			

Statistics for Table of ppnet by q1_dummy

Statistic	DF	Value	Prob
Chi-Square	1	1.1747	0.2784
Likelihood Ratio Chi-Square	1	1.1646	0.2805
Continuity Adj. Chi-Square	1	1.0057	0.3159
Mantel-Haenszel Chi-Square	1	1.1735	0.2787
Phi Coefficient		0.0344	
Contingency Coefficient		0.0344	
Cramer's V		0.0344	

Effective Sample Size = 994.09868015
Frequency Missing = 5.9011999965

Model Information		
**Data Set**	WORK.PAY	
**Response Variable**	q1_dummy	
**Number of Response Levels**	2	
**Weight Variable**	wmid1	Numeric for wmid1
**Model**	Binary Logit	
**Optimization Technique**	Fisher's Scoring	
**Variance Adjustment**	Degrees of Freedom (DF)	

Variance Estimation	
**Method**	Taylor Series
**Variance Adjustment**	Degrees of Freedom (DF)

**Number of Observations Read**	1005
**Number of Observations Used**	1000
**Sum of Weights Read**	999.9999
**Sum of Weights Used**	994.0987

Response Profile			
**Ordered Value**	**q1_dummy**	**Total Frequency**	**Total Weight**
**1**	important	633	634.76433
**2**	unimportant	367	359.33435

Probability modeled is q1_dummy='unimportant'.

Note: 5 observations were deleted due to missing values for the response or explanatory variables.

Class Level Information				
**Class**	**Value**	**Design Variables**		
**ppgender**	**female**	1		
	**male**	0		
**age4**	**18-34**	1	0	0
	**35-49**	0	1	0

Class Level Information				
**Class**	**Value**	**Design Variables**		
	**50-64**	0	0	1
	**65+**	0	0	0
**income4**	**$25-49.9K**	1	0	0
	**$50-74.9K**	0	1	0
	**$75K+**	0	0	0
	**<$25K**	0	0	1
**region**	**Midwest**	1	0	0
	**Northeast**	0	0	0
	**South**	0	1	0
	**West**	0	0	1

Model Convergence Status
Convergence criterion (GCONV=1E-8) satisfied.

Model Fit Statistics		
**Criterion**	**Intercept Only**	**Intercept and Covariates**
**AIC**	1302.794	1318.501
**SC**	1307.702	1372.486
**-2 Log L**	1300.794	1296.501

Testing Global Null Hypothesis: BETA=0			
**Test**	**Chi-Square**	**DF**	**Pr > ChiSq**
**Likelihood Ratio**	4.2931	10	0.9332
**Score**	4.3120	10	0.9322
**Wald**	3.9758	10	0.9484

Type 3 Analysis of Effects			
Effect	DF	Wald Chi-Square	Pr > ChiSq
ppgender	1	0.0716	0.7890
age4	3	2.6566	0.4477
income4	3	0.4401	0.9318
region	3	0.9030	0.8247

Analysis of Maximum Likelihood Estimates						
Parameter		DF	Estimate	Standard Error	Wald Chi-Square	Pr > ChiSq
Intercept		1	-0.5370	0.2555	4.4191	0.0355
ppgender	female	1	-0.0375	0.1403	0.0716	0.7890
age4	18-34	1	0.2708	0.2192	1.5261	0.2167
age4	35-49	1	0.0268	0.2350	0.0130	0.9092
age4	50-64	1	0.0493	0.2246	0.0481	0.8264
income4	$25-49.9K	1	-0.0541	0.1889	0.0820	0.7746
income4	$50-74.9K	1	0.0600	0.1839	0.1063	0.7444
income4	<$25K	1	-0.0791	0.2179	0.1319	0.7165
region	Midwest	1	-0.1768	0.2203	0.6439	0.4223
region	South	1	-0.0685	0.2060	0.1107	0.7394
region	West	1	-0.1629	0.2220	0.5380	0.4633

Odds Ratio Estimates			
Effect	Point Estimate	95% Wald Confidence Limits	
ppgender female vs male	0.963	0.732	1.268
age4 18-34 vs 65+	1.311	0.853	2.015
age4 35-49 vs 65+	1.027	0.648	1.628
age4 50-64 vs 65+	1.051	0.676	1.631
income4 $25-49.9K vs $75K+	0.947	0.654	1.372
income4 $50-74.9K vs $75K+	1.062	0.741	1.522
income4 <$25K vs $75K+	0.924	0.603	1.416
region Midwest vs Northeast	0.838	0.544	1.291

Odds Ratio Estimates			
Effect	Point Estimate	95% Wald Confidence Limits	
region South vs Northeast	0.934	0.624	1.398
region West vs Northeast	0.850	0.550	1.313

Association of Predicted Probabilities and Observed Responses			
Percent Concordant	52.1	Somers' D	0.066
Percent Discordant	45.5	Gamma	0.068
Percent Tied	2.3	Tau-a	0.031
Pairs	232311	c	0.533
